# An evaluation of superhydrophilic surfaces of dental implants - a systematic review and meta-analysis

**DOI:** 10.1186/s12903-019-0767-8

**Published:** 2019-05-10

**Authors:** Arkadiusz Makowiecki, Jakub Hadzik, Artur Błaszczyszyn, Tomasz Gedrange, Marzena Dominiak

**Affiliations:** 1 Armadent Private Practice, Kraków, Poland; 2 Department of Dental Surgery, Wroclaw Medical University, ul. Krakowska 26, 50-425 Wrocław, Poland; 3 Department of Oral Implantology, Wroclaw Medical University Wrocław, ul. Krakowska 26, 50-425 Wrocław, Poland; 4 Department of Orthodontics, Carl Gustav Carus Campus, Technische Universität Dresden, Germany, Fetscherstr. 74, 01307 Dresden, Germany

**Keywords:** Straumann SLActive, Thommen Inicell, Prospective studies, Randomized clinical trial, Bone loss, Alveolar bone loss, Complication, Success rate, Marginal bone loss, Stability

## Abstract

**Background:**

The characteristics of a dental implant surface have a decisive influence on the process of osseointegration. According to the current state of knowledge, surface modification can not only affect the morphology of cells, and in this way have a positive impact on osseointegration.

**Methods:**

The objective of this study was to compare survival rates and marginal bone loss as well as assess the degree of stability of Straumann SLAactive® and Thomenn Incell® implants with a superhydrophilic surface. Authors present review of data published between 01.01.2008 and 12.31.2016 that was found in PubMed/MEDLINE internet database, An Internet search of databases produced a total of 1230 studies, 20 publications were finally selected for the present study based on the established selection and exclusion criteria.

**Results:**

The statistical analysis was performed. A Cumulative Implant Survival Rate (CSR%) was 98.5%, Marginal bone loss (MBL) after 6 months was M = 0.60 mm and 0.6 5 mm after 12 months and secondary stability in a group Thommen implants M = 71.3 ISQ and M = 75.2 ISQ in group of Straumann.

**Conclusion:**

Despite certain differences in the values of the studied parameters, both of the systems, i.e. Thommen Inicell and Straumann SLActive, demonstrated a high survival rate, a high level of implant stability and low marginal bone loss.

## Background

Because of the current excellent success rate of implant treatment (as high as 99% [1]) and good aesthetics achievable using this method, this is no longer the only criterion that is considered when choosing an implant system. The dental implant industry is constantly seeking the most effective and safest connection between the implant and the prosthetic superstructure to prevent the marginal bone loss, and we also observe the evaluation of implant surfaces, especially those being in contact with the bone. In the search for the ideal implant researchers have considered a number of factors, such as: the position of the implant platform in relation to the height of the alveolar crest (supracrestal, bone level, subcrestal); the type of connection between the implant and the superstructure, e.g. utilizing the platform switching [2] or platform matching, [3]; the type of connection between the abutment and the implant where a Morse taper [4, 5], tri-channel, external-hex or internal-hex [6, 7] connection can be used; as well as, finally, the choice of implant neck, which can be polished or have a modified surface like the remaining part of the implant [8–10]. The implant systems available on the market combine these concepts in different ways.

The characteristics of the surface of a dental implant have a decisive influence on the process of osseointegration. The physical properties of implants, especially their surface characteristics, are responsible for the progress made in implant treatment in the last few decades. According to the current state of knowledge, modification of the microsurface can not only increase the surface but also affect the morphology of cells, and in this way have a positive impact on osseointegration [11, 12]. Many methods exist for increasing the implant surface roughness. One technique that is widely employed involves combining sand-blasting and etching with acid (SLA), and successful osseointegration using this approach has been widely documented in the literature. Through successive modifications of the surface, such as improving wettability and the hydrophilic properties of the implant, it is possible to further shorten the time needed to achieve secondary stability of the implant and accelerate absorption of proteins on the implant surface, which makes its surface hydrophilic.

Straumann [Straumann Holding AG, Switzerland] has developed an implant with a SLAactive® surface which has hydrophilic properties and is chemically active. Thommen **[**Thommen Medical AG, Switzerland**]** has devised an implant called Inicell®, whose hydrophilic surface accelerates the absorption of proteins on implant surfaces. Inicell is the conditioned state of the sandblasted and thermal acid-etched Thommen implant surface. During conditioning the surface chemistry of the microrough surface is slightly modified. Conditioning occurs immediately before implantation through contact with the conditioning agent (patent pending). The result of this process is increased surface energy and improved wettability due to superhydrophilic properties. Both types of implant were developed with the aim of significantly shortening the time required to achieve biological secondary stability (osseointegration) and reduce healing time to 3–4 weeks [13–15].

The objective of this study was to compare survival rates and marginal bone loss as well as assess the degree of stability of dental implant with superhydrophilic surface - Straumann SLAactive® and Thomenn Incell® implants. The present article systemises the data from available publications and in this way provides a summary of the available information on both the purpose and effectiveness of the above-mentioned implants.

## Methods

### Choice of publications

The articles were sought on the PubMed/MEDLINE internet database. For an article to be included in the analysis it had to be an English language publication dated between 01.01.2008 and 12.31.2016 in the case of Straumann implants or between 01.01.2010 and 12.31.2016 in the case of Thommen implants. The review employs the PRISMA statement.

A word search was conducted based on the following key terms:

“Thommen”, “Straumann”, “Straumann SLActive”, “Thommen Inicell”, “prospective Studies”, “clinical Trial”, “randomized Clinical Trial”, “bone loss”, “alveolar bone loss”, “complication”, “success rate”, “failures”, “marginal bone loss”, “stability”, “patient satisfaction”.

The topic of the research in the analyzed articles had to be either Straumann SLActive® implants or Thommen Inicell® implants.

The authors based their search strategy on the PICO model (P – Problem, I – Intervention(s), C – Comparison, O – Outcome).

- Problem:

A comparison of survival rates and marginal bone loss as well as an assessment of the degree of stability of dental implants with superhydrophilic surfaces - Straumann SLAactive® and Thomenn Incell® implants.

- Intervention;

The articles searched on the PubMed/MEDLINE internet database were selected on the basis of the established criteria.

- Comparison:

A comparison and analysis of the results described in the selected studies.

- Outcome:

An assessment of the results.

In the first phase of the study the authors analysed titles and abstracts with the aim of assessing whether the material contained in them meets the conditions for further analysis. The selection criteria described below were applied.

### Selection criteria

- Prospective clinical studies on patients fitted with Straumann SLActive® or Thommen Inicell® implants, or with both these systems
- Studies which describe both the criteria of success and failure
- No surgical techniques, types of restoration, age, sex, etc. were distinguished

### Exclusion criteria

- Studies on animals
- Studies describing the placement of > = 10 implants
- Studies in which the follow-up time for a prosthetic restoration was less than 6 months
- Retrospective studies
- Publications of individual clinical case studies
- Short communications

### Data search

The selection criteria and exclusion criteria provided the basis for selecting articles with the aim of analysing their full content. The selected articles contained such information as the following: the type of study conducted, the number of implants, the length and diameter of the implants, the name of the implant system, the type of prosthetic restoration, the loading protocol for implants, the type of implant-superstructure connection, information on follow-ups both during the healing process and after loading (min 6 months), measurements of marginal bone loss (MBL) in the alveolar ridge, assessments of implant stability at different stages, information on any possible complications as well as implant loss, and information regarding patient satisfaction.

The studies described in the papers were not analyzed with regard to the influence of ethnic factors and their impact on the research results, because the authors did not adopt such criteria and data of this type were not presented.

In those cases where full data is missing attempts were made to contact the authors of the articles via mail. The failure of an author to respond did not constitute a basis for rejecting the article. However, in such a case, the data used was incomplete.

### Study methods

The data obtained from the selected articles has been gathered in Tables 1 and 2. The studies described in the literature were assessed in terms of their impact on the conducted meta-analysis. A Cumulative Implant Survival Rate (CSR%) of no less than 95% was deemed necessary to draw valid conclusions. The analyses were based on the following tests: the Student’s t test and the Pearson’s chi-squared test of independence.

**Table 1:** Analysed publications and Cumulative Implant Survival Rate (CSR%)

Study	Year of pub.	No. implants	Location	Type of restoration	Protocol of implantation	Follow-up period in months	Number of lost implants	CSR in %
Eekeren et al. [16]	2015	78	posterior maxilla and mandible	PFM crowns	one-stage	12	2	97
Hasson et el. [17]	2014	77	posterior maxilla and mandible	PFM and provisional crowns	one-stage	24	0	100
Held et al. [18]	2013	34	posterior maxilla and mandible	PFM crowns	two-stage	16	1	97
Hicklin et.al. [19]	2015	20	posterior maxilla	provisional crowns	two-stage	6	0	100
Hinkle et al. [20]	2014	24	posterior maxilla and mandible	provisional crowns	one-stage	12	1	96
Le Gac et al. [21]	2015	1337	maxilla and mandible	all Types	one- and two-stage	24–60	7	99.5
Liaje et al. [22]	2013	23	maxilla and mandible	PFM crowns	one-stage	12	0	100
Yu et al. [23]	2016	20	maxilla	PFM crowns	one-stage	12	0	100
Hadzik et al. [11]	2017	16	mandible	PFM crowns	two-stage	9	0	;
Dard et al. [24]	2016	75	maxilla and mandible	PFM crowns	two-stage	12	0	100
Allen et al. [25]	2016	20	maxilla	PFM crowns	two-stage	24	0	100
Al-Nawas et al. [26]	2012	160	mandible	locators	one-stage	12	3	98
Calvo-Guirado et al. [27]	2016	40	mandible	PFM crowns	two-stage	12	1	97.5
Ganales et al. [28]	2008	383	maxilla and mandible	PMF crowns, composite on metal	one-stage	12	10	97
Markovic et al. [29]	2016	40	mandible	locators	two-stage	12	0	100
Morton et al. [30]	2010	89	maxilla and mandible	PFM and provisional crowns	one-stage	24	3	97
Nedir et al. [31]	2013	37	maxilla	PFM crowns	two-stage	12	2	94
Nicolau et al. [32]	2013	383	maxilla and mandible	PFM and provisional crowns	one-stage	36	11	97
Ryu et al. [33]	2016	30	maxilla and mandible	PFM and provisional crowns	two-stage	13	0	100
Slotte et al. [34]	2012	94	mandible	PFM crowns	two-stage	24	4	96

**Table 2:** Marginal bone loss (MBL), Primary and Secondary Implant Stability in Ostell ISQ values

Study	Year of publication	MBL at 6 months	MBL at 12 months	Primary stability. Ostell ISQ	Secondary stability. Ostell ISQ	Biologic complacations
Eekeren et al. [16]	2015	not reported	0.4 ± 0.5	not reported	not reported	1
Hasson et el. [17]	2014	not reported	0.9 ± 0.5	not reported	not reported	0
Held et al. [18]	2013	1.46 ± 0.7	not reported	43 ± 9	68 ± 10	2
Hicklin et.al. [19]	2015	0.97	not reported	78	80	0
Hinkle et al. [20]	2014	1.10 ± 0.58	0.99 ± 0.29	not reported	not reported	1
Le Gac et al. [21]	2015	not reported	not reported	not reported	not reported	7
Liaje et al. [22]	2013	0.14 ± 0.05	0.20 ± 0.40	78.39 ± 3.95	76.17 ± 4.45	0
Yu et al. [23]	2016	not reported	0.35 ± 0.60 (24 months)	not reported	not reported	0
Hadzik et al. [11]	2017	0.53 ± 0.2 (3 months)	0.57 ± 0.3 (9 months)	66.5 ± 16.5	73.9 ± 8.4	0
Dard et al. [24]	2016	0.53 ± 0.6	0.74 ± 0.68	not reported	not reported	0
Allen et al. [25]	2016	not reported	0.81 24 months	68.04	74.63	0
Al-Nawas et al. [26]	2012	0.23 ± 0.37	0.32 ± 0.55	not reported	not reported	3
Calvo-Guirado et al. [27]	2016	0.64 ± 0.38	0.71 ± 0.11	75.22 ± 1.23	78.33 ± 1.76	1
Ganeles et al. [28]	2008	not reported	0.77 ± 0.93	not reported	not reported	10
Markovic et al. [29]	2016	not reported	not reported	71.22 ± 6.05	67.68 ± 3.27	0
Morton et al. [30]	2010	0.25	0.21	not reported	not reported	3
Nedir et al. [31]	2013	not reported	0.5 ± 0.75	not reported	not reported	2
Nicolau et al. [32]	2013	0.69 ± 0.80	0.76	not reported	not reported	11
Ryu et al. [33]	2016	not reported	0.98 ± 88	76.34 ± 5.89	81.62 ± 2.00	0
Slotte et al. [34]	2012	not reported	0.43	not reported	not reported	4

### Statistical analysis

The statistical analysis was performed using STATISTICA v. 12 [StatSoft Polska Sp. z o.o. Poland]. Pearson chi-square i and Mann*–*Whitney U nonparametric test were conducted. All data were given as means ± standard deviation (SD).

## Results

### Study characteristics

**Fig. 1:**
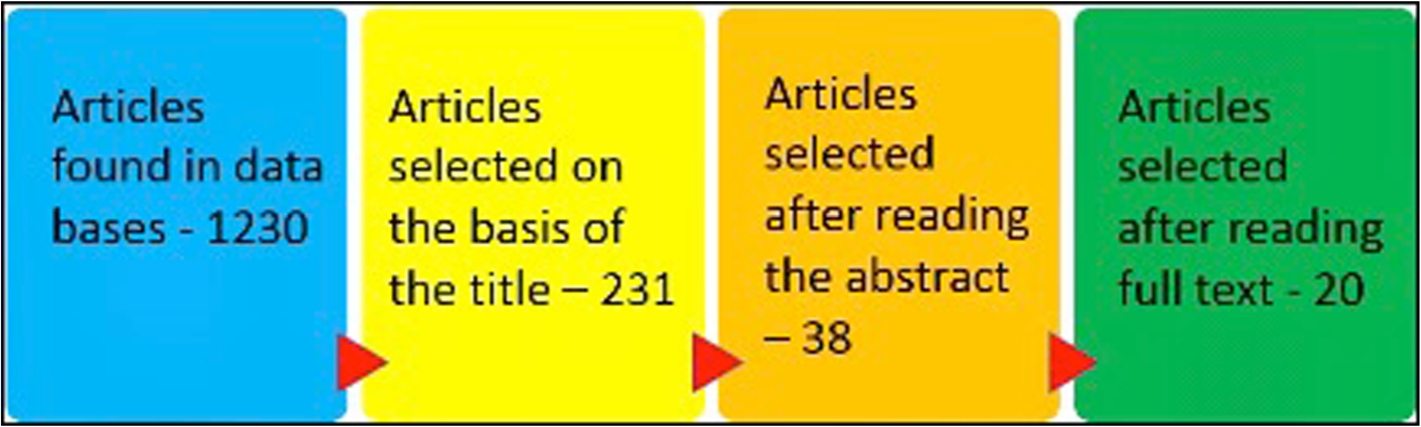
Articles selected for analysis

An Internet search of databases produced a total of 1230 studies concerning Straumann SLActive® and Thommen Inicell® implants with a superhydrophilic surface: 178 publications on Thomenn Incell® implants and 1052 on Strauman SLAactive® implants. At the outset, 999 publications were excluded from the analysis, and of the remaining 231 abstracts 38 articles were selected for full analysis. A total of 20 publications were finally selected for the present study based on the established selection and exclusion criteria (Fig. 1).

**Fig. 2:**
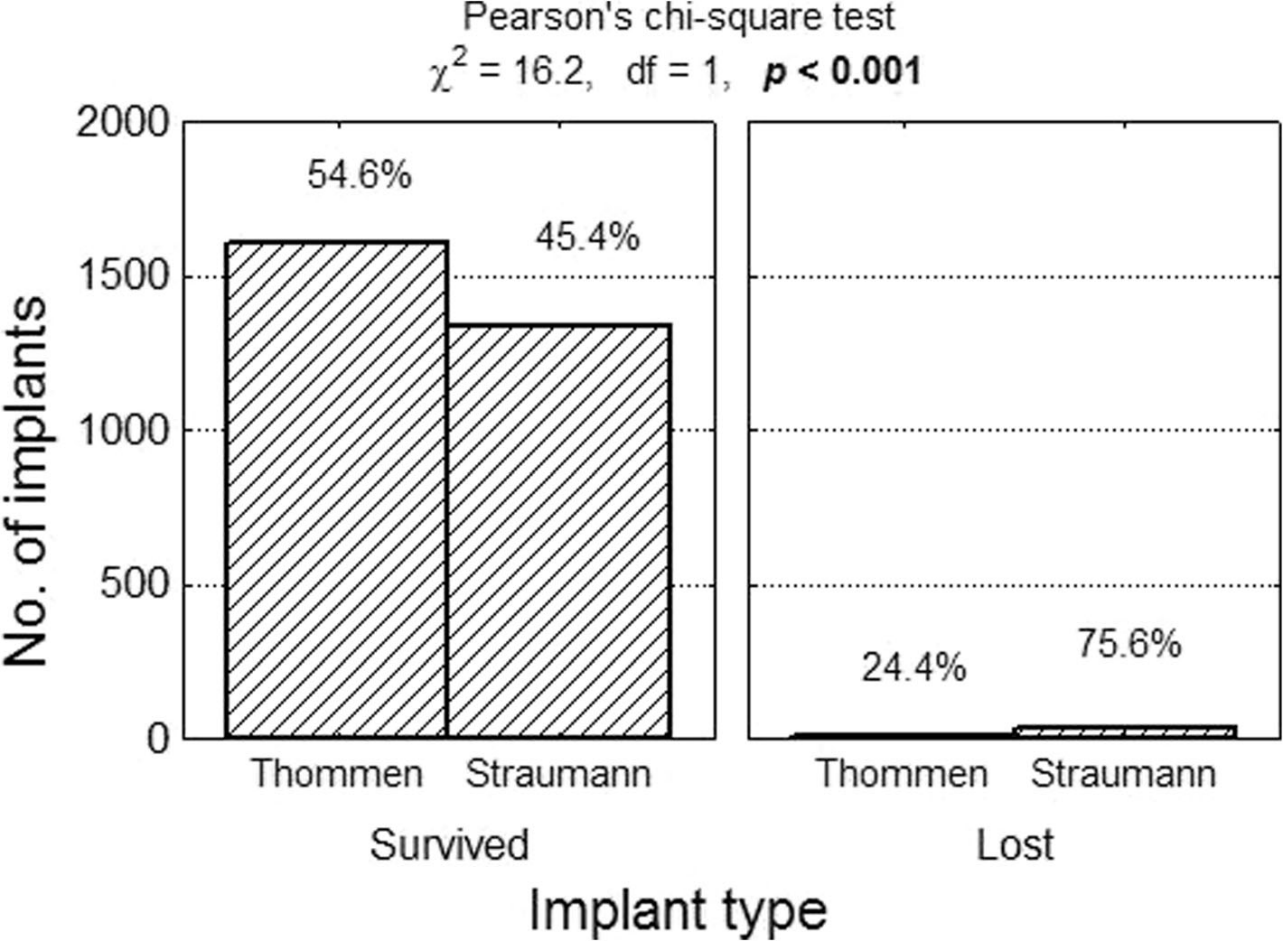
Number (percentage) of implants in groups differing from one another in terms of survival rate test of independence result

**Fig. 3:**
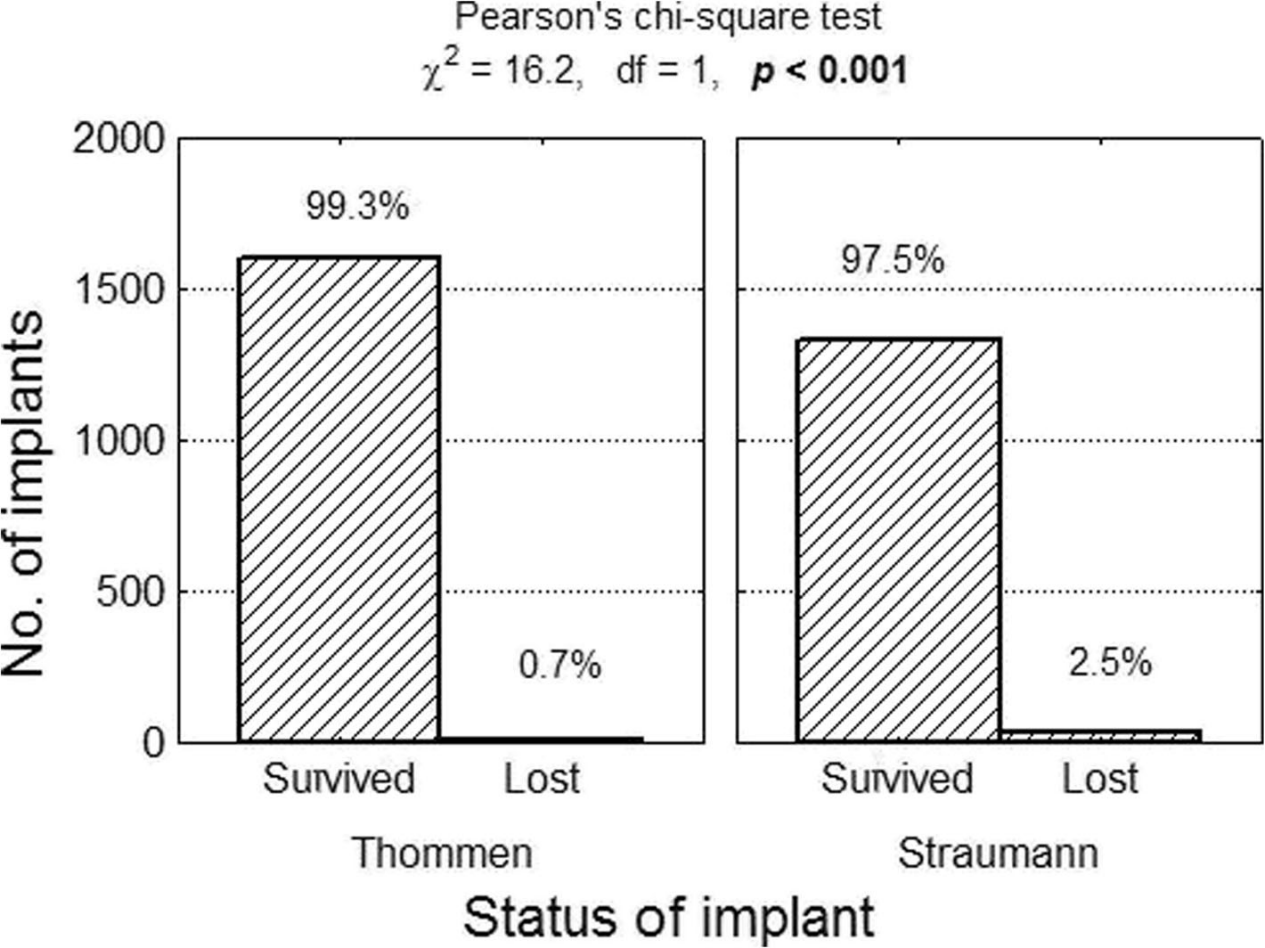
Number (percentage) of implants in groups differing from one another in terms of implant type test of independence result

In the 20 publications which qualified for the analysis based on the selection criteria the implant survival rates ranged from 94.6 to 100.0%. A total of 2890 implants were evaluated (1613 Thommen implants and 1367 Straumann implants). Forty-five implants from this study population were lost, which means that the overall implant survival rate (CSR) was 98.5% (Thommen CSR = 99.3%; Straumann CSR = 97.5%). The risk of implant loss in the case of the Thommen implants was 0.007 (11/1613), while the corresponding risk for Straumann implants amounted to 0.025 (34/1367). The relative risk of implant loss in the case of the Straumann implants compared with the Thommen implants was RR = 0.025/0.007 = 3.65 (a 95% confidence interval: from 1.86 to 7.17), which means the risk was more than three and a half times greater (Figs. 2, 3).

### Marginal bone loss (MBL)

Of the 20 analysed reports (articles) complete information (mean and standard deviation) on marginal bone loss after 6 and 12 months was only found in 9 (MBL after 6 months) and 13 (MBL after 12 months) cases, respectively. The mean MBL after 6 months (in a group comprising 755 implants) was M = 0.60 mm while the standard deviation (SD) was 0.81 mm. The mean MBL after 12 months (in a group comprising 963 implants) amounted to M = 0.65 mm while the standard deviation (SD) = 0.85 mm.

**Fig. 4:**
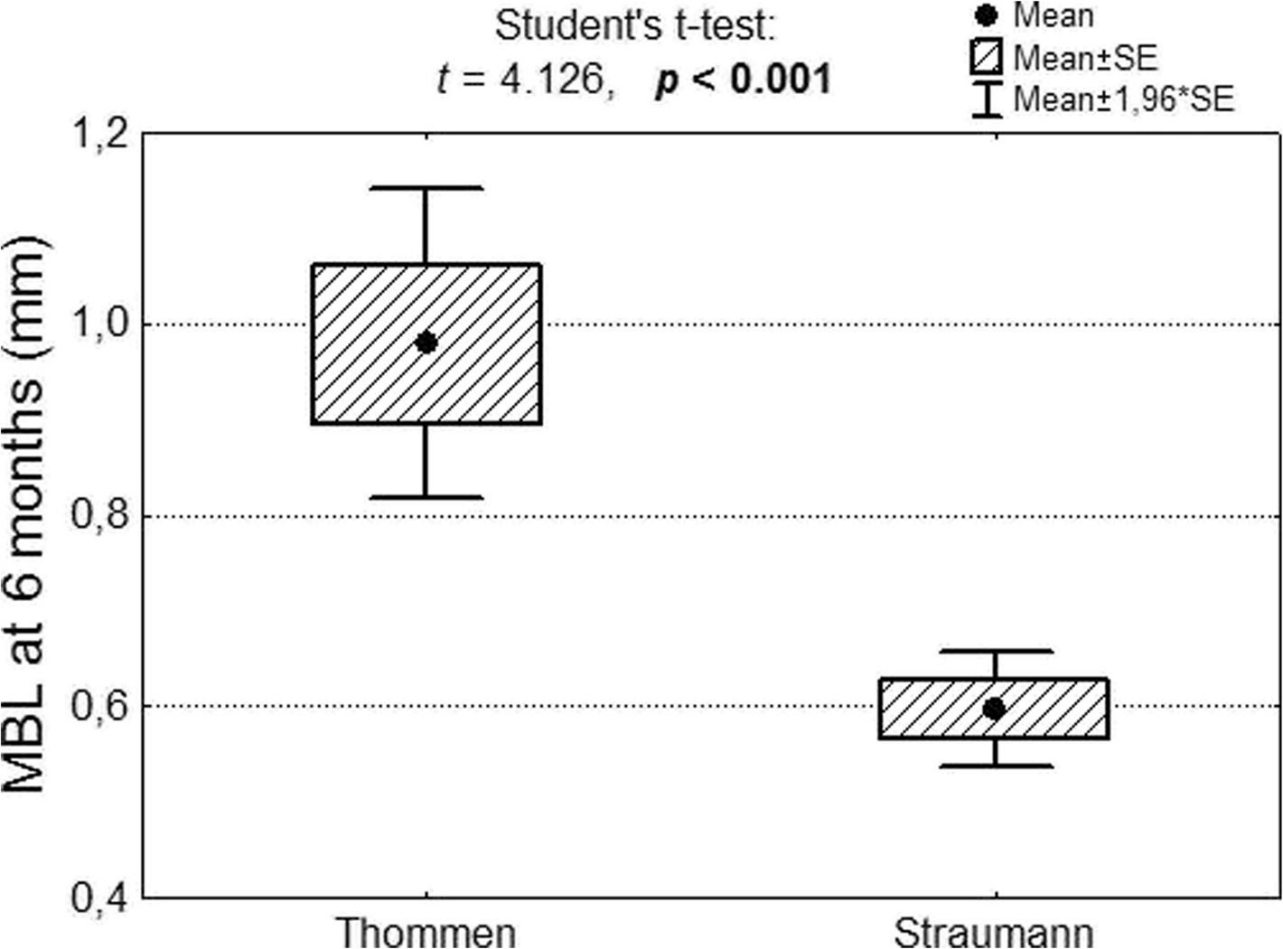
Marginal bone loss after 6 months in the case of Thommen and Straumann implants as well as significance test results

After 6 months, the mean marginal bone loss in a group of 81 Thommen implants was M = 0.98 mm; SD – 0.74 while the mean bone loss in a group comprising 674 Straumann implants was M = 0.56 mm; SD = 0.82. The marginal bone loss after 6 months was significantly greater in the Thommen implant group (Fig. 4).

**Fig. 5:**
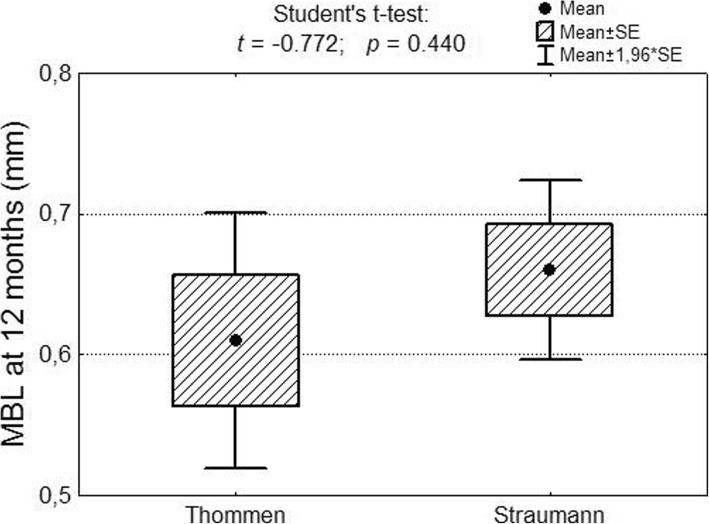
Marginal bone loss after 12 months in the case of Thommen and Straumann implants as well as significance test results

After 12 months, the mean marginal bone loss in a group comprising 222 Thommen implants was M = 0.61 mm; SD – 0.69, while the mean bone loss in a group comprising 741 Straumann implants amounted to M = 0.66 mm; SD = 0.89. No significant differences in marginal bone loss after 12 months were observed in either group of implants (Fig. 5).

### Assessment of primary and secondary stability

Only 6 reports provided complete information on primary stability. Two studies concerned Thommen implants (a total of 57 implants) while the other 4 discussed on Straumann implants (126 implants). The mean primary stability (in a group comprising 183 implants) was M = 68.2, and the standard deviation SD was 7.4. Secondary stability was also assessed in these studies. The mean primary stability (in the group of 183 implants) was M = 74.0, and the standard deviation SD was 5.6.

**Fig. 6:**
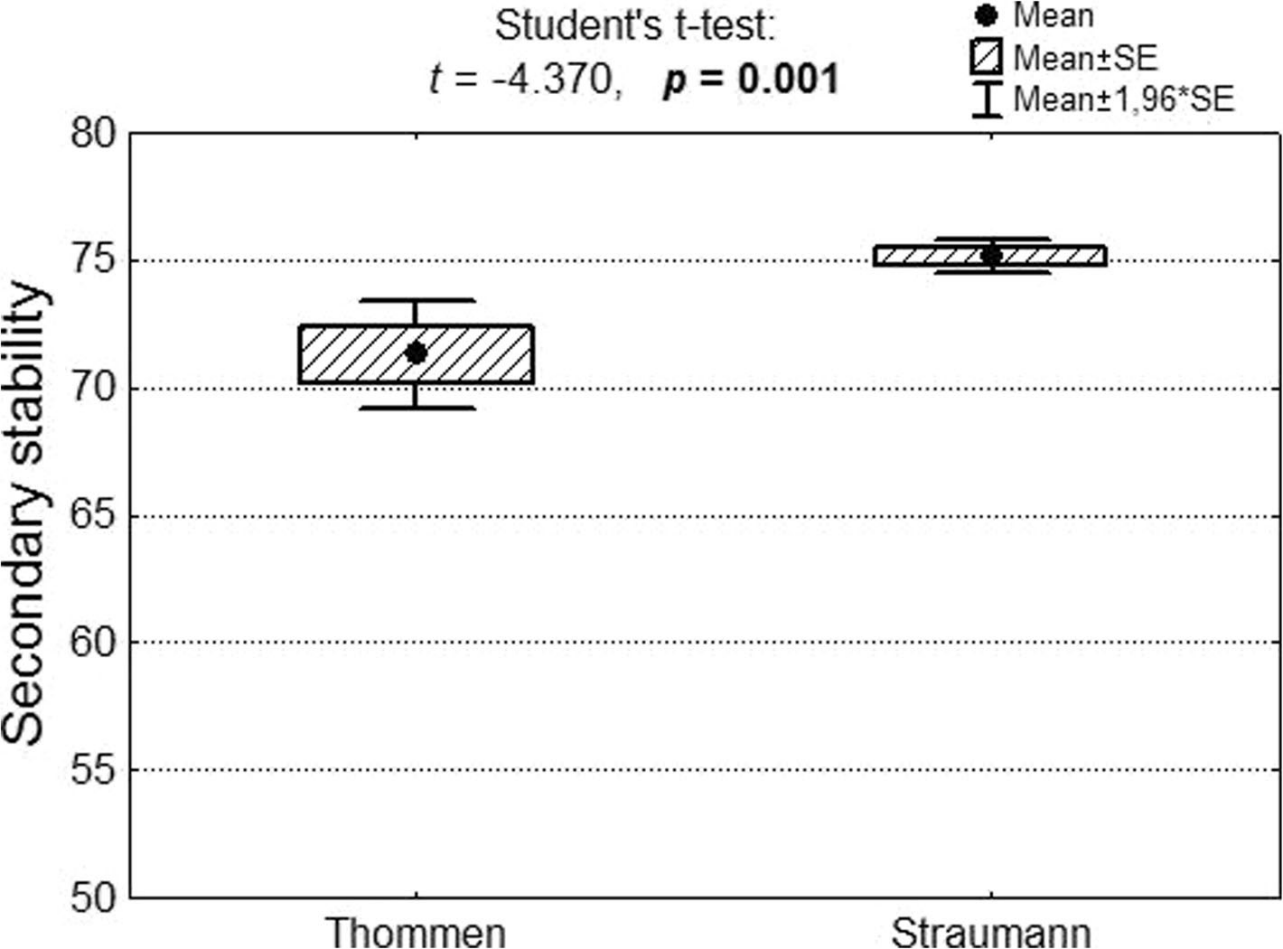
Secondary stability of Thommen and Straumann implants as well as results of significance test

Primary stability in a group comprising 57 Thommen implants amounted to M = 57.3; SD = 7.4, while in a group comprising 126 Straumann implants primary stability was M = 73.1; SD = 7.4. Primary stability measured on the ISQ Osstell scale was significantly higher in the Straumann implant group (Fig. 6).

**Fig. 7:**
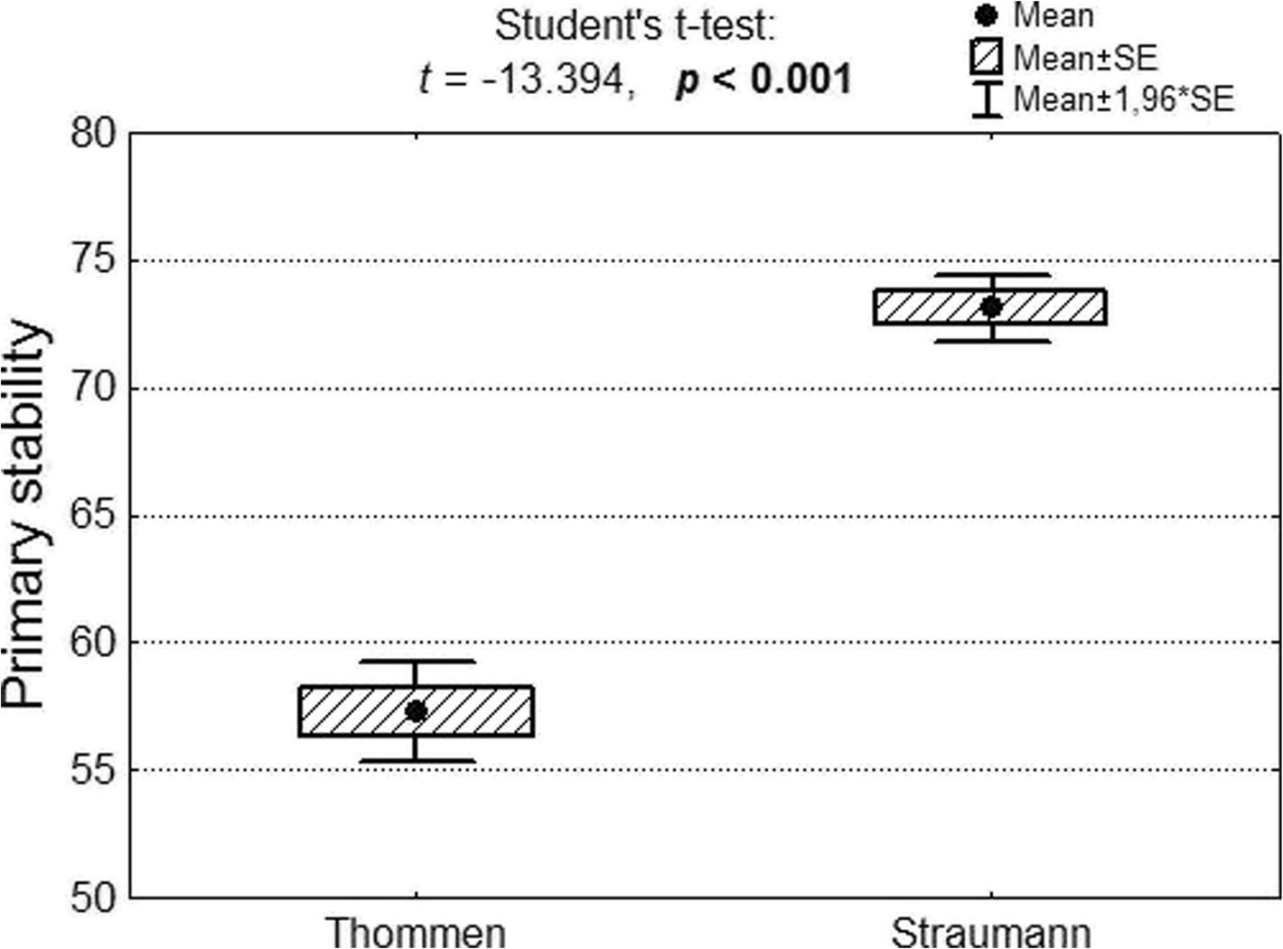
Primary and secondary stability of Thommen and Straumann implants as well as results of significance test

Secondary stability in a group comprising 57 Thommen implants amounted to M = 71.3; SD = 8.2, while in group of 126 Straumann implants the corresponding value was M = 75.2 mm; SD = 3.8. Secondary stability was still greater in the Straumann group (Fig. 7).

### Biological complications

**Fig. 8:**
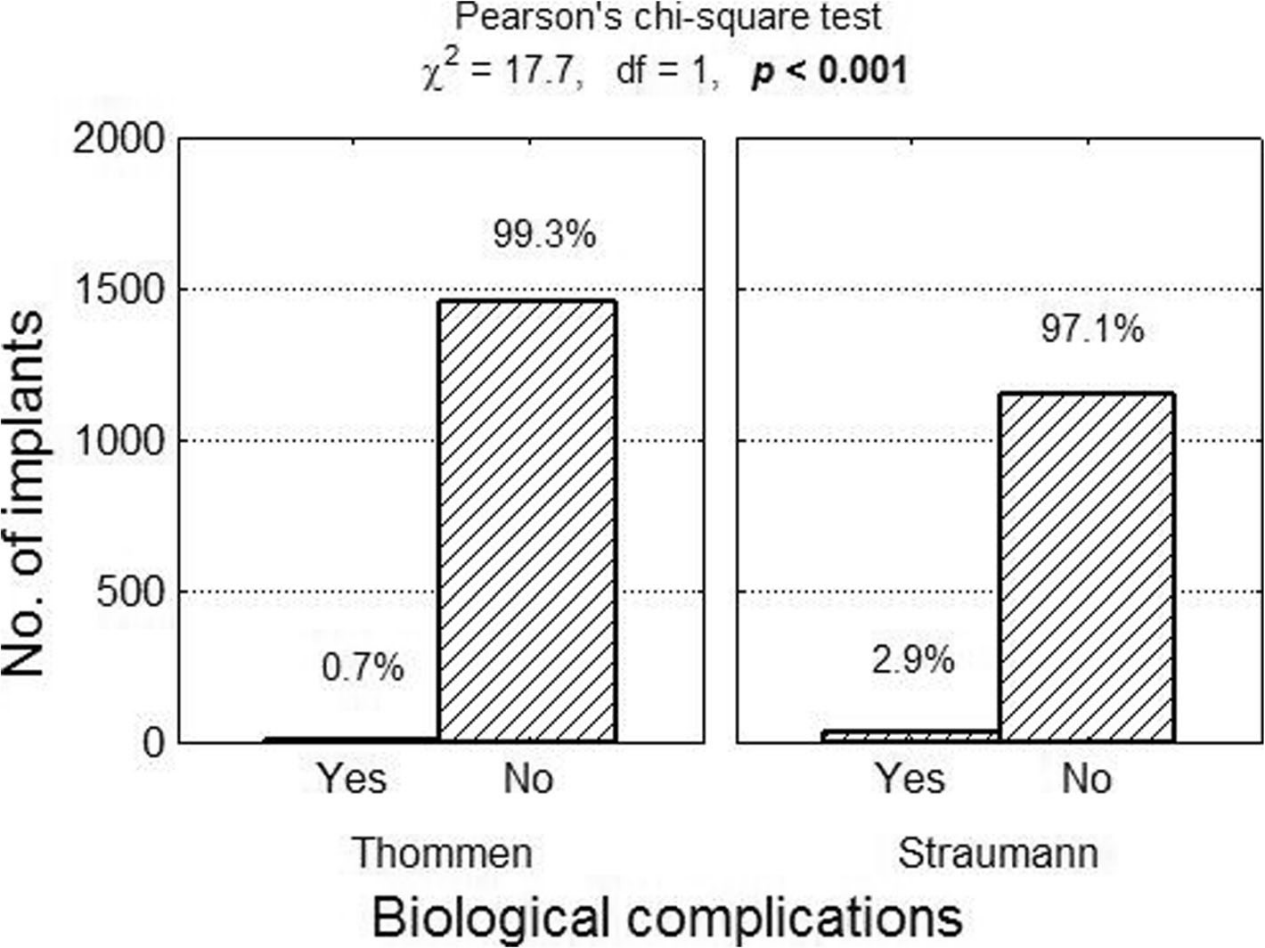
Number (percentage) of implants in groups differing from one another in terms of biological complications test of independence result

**Fig. 9:**
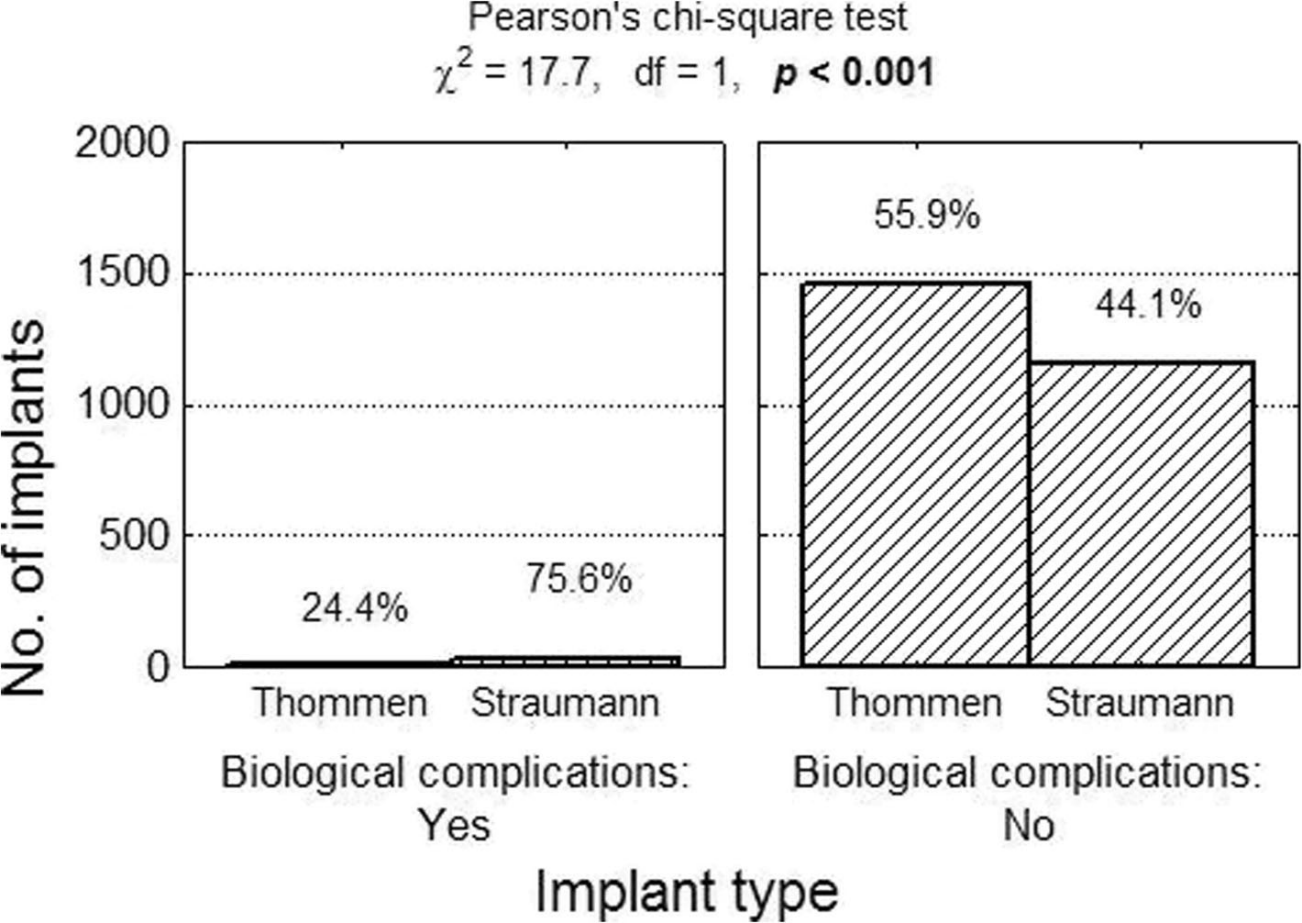
Biological complications of Thommen and Straumann implants as well as result of test of independence

The following were classified as biological complications: loss of implant osseointegration as a result of infection, improper hygiene (accumulation of bacterial plaque), and peri-implantitis. Information on the number of biological complications was found in 11 articles (4 reports concerned Thommen implants and 7 discussed Straumann implants). In total, there were 45 reported complications in a group comprising 2659 implants (1.69%). Among Thommen implants biological complications occurred in 0.75% of cases (11 complications in 1473 implants), while 2.87% of Straumann implants (34 out of 1186 implants) were affected. The risk of biological complications in the case of Thommen implants was 0.007 compared with 0.028 for Straumann implants. The relative risk of biological complications in the case of Straumann implants compared with Thommen implants was RR = 0.028/0.007 = 3.76 (95% confidence interval: from 1.91 to 7.39), which means it was more than three and a half times greater (Figs. 8 and 9).

### Mechanical complications

The following were classified as mechanical complications: all forms of damage to the implant-superstructure complex necessitating the removal of the implant.

None of the 20 articles provided any information on mechanical complications.

### Aesthetic complications

In none of the articles analysed in this study did the authors report any problems of an aesthetic nature. Neither white aesthetics (the shape, colour etc. of the prosthetic restorations) nor pink aesthetics (the architecture of the emergence profile and gums in the region of the placed implant) were assessed.

In a clear majority of cases the researchers did not assess the satisfaction of patients with the treatment they received.

## Discussion

The primary objective of the present study was to assess the basic success parameters for Straumann SLActive® and of Thommen Inicell® implants with a superhydrophilic surface after either functional immediate loading or loading after a short healing period (up to 4 weeks).

Straumann [Straumann Holding AG, Switzerland] has developed an implant with a new kind of surface that has hydrophilic properties and is chemically active. The SLActive® surface differs from the SLA surface due to its hydrophilic properties. Its initial angle of contact with water is 00, compared with 139.90 for SLA. Preparation of such a surface involves sandblasting it with Al_2_O_3_ abrasive grain with 25–50 μm particle size and then etching it with a mixture of HCl + H_2_SO_4_. After etching the implants are rinsed in a nitrogen atmosphere and then stored in a NaCl solution. A noble gas atmosphere followed by physiological salt protects the titanium surface from contamination with hydrocarbon and carbon compounds originating from atmospheric air. The aim of such chemical modifications is to maintain the hydrophilic character and the high natural surface energy of titanium dioxide up to the moment of implant placement. The SLActive surface shown in the SEM image is identical to the SLA type surface [35].

The Thommen Incell® **[**Thommen Medical AG, Switzerland] implants feature a modified surface containing active hydroxyl ions (OH-) which increase the surface energy of implant and accelerate the absorption of proteins on the implant surface, thereby making the surface hydrophilic. The articles analysed in the study differed from one another in terms of the type of research protocol adopted (no augmentation, augmented bone, sinus lift, etc.), the type of prosthetic restoration used, the data assessed, the dates as well as the definitions used, the diameter and length of the implants placed (from 4 mm up to 14 mm) as well as the total study period (from 6 to 60 months). The parameters assessed were those which were most commonly repeated in the articles included in the research.

According to the authors of the evaluated studies, the most important criterion of success is the Cumulative Implant Survival Rate (CSR%), as it is the only parameter which was assessed and described in each analysed publication. The CSR rate varied between 94% [31] and 100% [11, 17, 19, 22–25, 29, 33]. Of the 2890 observed implants (1613 Thommen implants and 1367 Straumann implants) a total of 45 were lost, which gives a CSR of 98.5%. This value is fully comparable with the results of studies based on standard procedures [36, 37].

The second parameter most commonly evaluated by researchers was MBL - Marginal Bone Loss. Of the 20 publications analysed in this study 9 provided complete data on marginal bone loss after 6 months (MBL after 6 months), while 13 described marginal bone loss after 12 months (MBL after 12 months) (Tab. 2). Based on the available data average marginal bone loss (MBL) was M = 0.60 mm after 6 months and M = 0.65 mm after 12 months. A comparison of the results obtained with the results achieved with standard procedures shows that they do not deviate from the established norm, according to which MBL should not be greater than 1.5 mm in the first year and then 0.1 mm in each following year [38–41]. It is important to note that some researchers claim that original remodelling of the alveolar process occurs after implant loading with the aim of restoring biological width, such that MBL should be assessed 12 months after restoration [42].

The third evaluation criterion was the assessment of stability. For the needs of our analysis we exclusively used results obtained using an Osstell measuring device (Osstell AB, Goteborg, Sweden). Osstell measures stability by analysing the RFA resonance frequency (Resonance Frequency Analysis), which provides us with objective information on the degree of implant integration [43] independent of the researchers. The Implant Stability Quotient (ISQ) is read in RFA (Resonance Frequency Analysis), which is measured in kHz. The ISQ scale converts kHz values into a scale of 1 to 100 ISQ units, which is more practical for clinical assessment purposes. The result is given within a range of 1 to 100 ISQ units (Implant Stability Quotient). The higher the ISQ value the greater the stability of the implant. The frequency analysis technique makes it possible to assess implant stability as functions of implant-bone connection rigidity. The ISQ value depends on the bone density, implant healing time, the ratio of the exposed part of the implant to the part embedded in bone as well as the type of implant [44]. The mean primary stability was M = 68.2, the standard deviation SD = 7.4, mean secondary stability M = 74.0 and the standard deviation SD = 5.6. According to the protocol devised by Osstell, such values ensure safe implant loading. Eight of the 20 studies measured stability [11, 18, 19, 22, 25, 27, 29, 33]. Hicklin et al. and Allen et al. [19, 25] did not report any standard deviation values when measuring stability, and as a consequence these results were excluded from the analysis. In the other studies the researchers assessed stability in descriptive terms (e.g. correct, very good, etc.) or used a Periotest device.

Biological complications affected 45 of the 2659 placed implants, which translates into a complication rate of 1.69%. In the majority of cases, early implant loss was a consequence of complications in the osseointegration process, one reason for which may have been overheating of the implant bed, infection or micro movements in the case of immediate loading. More than 50% of cases of implant loss occurred at an early stage, i.e. before functional loading of the implant [45–47]. Late implant loss, as a result of bone loss caused by infection or peri-implantitis, occurred in approximately 60% of cases in the first year after loading. A study conducted by Snauwaert et al. showed that early implant loss as a result of biological complications occurred in 3.8% of cases, while late implant loss affected 2% of cases [48].

However, it is also important to bear in mind that the majority of researchers use as their measure of success the simple ratio of the total number of placed implants in relation to the total number lost. Such an approach is not entirely correct for it does not consider other factors that have an impact on the survival rate of implants, including [49]:

- factors connected with patients selection and their status: nicotine addiction, bruxism, diabetes, alcohol abuse
- factors connected with the implant placement procedure itself: primary stability, implant insertion torque, bone density, the position of the implant in the alveolar process, the implant loading protocol
- factors connected with the implant system used: surface type, length, diameter, design, type of connection between implant and superstructure
- factors connected with the prosthetic restoration: cemented restoration vs screw-retained restoration, individual crown vs a bridge or cantilever, fixed denture vs removable denture, occlusion, the material used (porcelain vs composite), ratio of crown height to implant length (C/1), the shape and position of the teeth or antagonistic restorations
- biological factors: assessment of periodontal tissue (PD – probing depth), attached gingiva, keratinized gingiva zone (HKT – height of keratinized tissue, TKT – thickness of keratinized tissue), accompanying periodontal diseases, patient hygiene level [50, 51].

However, considering the fact that analogous schema is used for conducting research when assessing standard implant procedures we can state that the above-described results fall within the accepted schema for studies addressing implant-prosthetic treatment issues.

In a clear majority of the studies cited here the authors did not assess the satisfaction of patients with the treatment they received. In those manuscripts which did discuss this issue no assessment was made using the Visual Analogue Scale. Hinkle et al. provided information on patient satisfaction in descriptive form: "All patients who participated in the study were completely satisfied [20]. Slotte et al. assessed patient satisfaction on the basis of a non-specific questionnaire, which was filled in by the patients on completion of the treatment [34]. In turn, when describing patient satisfaction following treatment Dard M. et al. observed that in cases of shorter treatment duration due to earlier implant loading patients had a more positive view of their experience than patients whose treatment followed the standard procedure [24].

## Conclusion

The assessment revealed certain differences between the two systems in terms of the studied parameters: the risk of implant loss was greater in the case of the Straumann implants compared with the Thommen implants. In turn, marginal bone loss (MBL) was higher after 6 months with the Thommen implants while primary and secondary stability was higher in the case of the Straumann implants. However, these differences were not significant enough to declare with any authority that one implant type has an advantage over the other. Based on the analysis and the implant placement success criteria devised by Buser et al. [52] (the implant – during functional loading – produces no pain or any kind of discomfort, there are no signs of inflammation, infection or implant mobility, and a radiological examination shows no low density bone structure foci around the implant or recurrent bone loss) we can state that both systems, Thommen Inicell and Straumann SLActive, help shorten the time of implant-prosthetic treatment while ensuring a high survival rate, good implant stability and low marginal bone loss.

Thommen

**Table:** 

Articles found in databases	178
Articles excluded on the basis of the title	63
Articles selected on the basis of the title	115
Articles excluded after reading the summary	104
Articles selected after reading the summary	11
Articles excluded after reading the full text	3
Articles selected after reading the full text	8


**Straumann**

**Table:** 

Articles found in databases	1052
Articles excluded on the basis of the title	936
Articles selected on the basis of the title	116
Articles excluded after reading the summary	84
Articles selected after reading the summary	27
Articles excluded after reading the full text	16
Articles selected after reading the full text	12

**Total: Thommen and Straumann**

**Table:** 

Articles found in databases	1230
Articles excluded on the basis of the title	999
Articles selected on the basis of the title	231
Articles excluded after reading the summary	188
Articles selected after reading the summary	38
Articles excluded after reading the full text	19
Articles selected after reading the full text	20

## Abbreviations

CSR%: Cumulative Implant Survival Rate
HKT: Height of keratinized tissue
ISQ: Implant Stability Quotient
MBL: Marginal bone loss (MBL)
PD: Probing depth
RFA: Resonance Frequency Analysis
SLA: Sand-blasting and etching with acid implant surface
TKT: Thickness of keratinized tissue
